# Integrating machine learning and blockchain to develop a system to veto the forgeries and provide efficient results in education sector

**DOI:** 10.1186/s42492-021-00084-y

**Published:** 2021-06-21

**Authors:** Dhruvil Shah, Devarsh Patel, Jainish Adesara, Pruthvi Hingu, Manan Shah

**Affiliations:** 1 Department of Computer Engineering, Vishwakarma Government Engineering College, Ahmedabad, Gujarat 382424 India; 2 Department of Chemical Engineering, School of Technology, Pandit Deendayal Energy University, Gandhinagar, Gujarat 382426 India

**Keywords:** Machine learning, Blockchain, Education

## Abstract

Although the education sector is improving more quickly than ever with the help of advancing technologies, there are still many areas yet to be discovered, and there will always be room for further enhancements. Two of the most disruptive technologies, machine learning (ML) and blockchain, have helped replace conventional approaches used in the education sector with highly technical and effective methods. In this study, a system is proposed that combines these two radiant technologies and helps resolve problems such as forgeries of educational records and fake degrees. The idea here is that if these technologies can be merged and a system can be developed that uses blockchain to store student data and ML to accurately predict the future job roles for students after graduation, the problems of further counterfeiting and insecurity in the student achievements can be avoided. Further, ML models will be used to train and predict valid data. This system will provide the university with an official decentralized database of student records who have graduated from there. In addition, this system provides employers with a platform where the educational records of the employees can be verified. Students can share their educational information in their e-portfolios on platforms such as LinkedIn, which is a platform for managing professional profiles. This allows students, companies, and other industries to find approval for student data more easily.

## Introduction

In the current age of technological innovation, various industries are embracing new technologies, providing them with further benefits. The educational discipline has shown an open-minded approach to such technological innovations and has embraced technologies best adapted for student development, such as the introduction of a massive open online course (MOOC), which has worked well for more productive student career growth. Technologies such as blockchain and machine learning (ML), which are showing tremendous development and potential in their respective fields, can be utilized in the educational discipline. The rise of digital currency bitcoin in recent years has drawn attention toward its base technology, called blockchain. It has the advantages of decentralization, a tamper-proof construction, a distributed ledger, and transparency of the accessibility and details. Therefore, blockchain technology has been observed in the fields of banking, education, supply chain management, and copyright protection, among others [1]. Blockchain is essentially like a permanent record book that has all transactions listed in chronological order [2]. All data are in the form of a long chain of connected data items that are stored on each participating computer and the inclusion of new data items is achieved only through a consensus of the majority of participants [3]. The ability of computers to achieve predictions from the experience and mimicking of the human brain has increased human curiosity in the field of ML and artificial intelligence [4]. According to Mitchell, professor of computer science and ML at Carnegie Mellon, a computer program is said to learn from experience E in relation to some function T and some output measure P, if its output on T, as calculated by P, increases with E. In such areas, the availability of different ML algorithms will offer a fruitful prediction by determining the correct pattern from the large number of data available for training [5]. This paper aims to demonstrate the use of ML and blockchain technology in the educational sector. Blockchain technology aims to avoid forgeries of student achievements and certifications [6]. In addition, it helps validate student data, which in turn addresses the main concern regarding security in the educational discipline [7]. The use of ML techniques for educational proposals is an evolving field that aims to establish methods to explore data from computational educational settings and to discover meaningful patterns [8]. The conventional way to store an achievement in the form of a certificate or degree for any student is through a centralized database system. As a problem with this centralized approach, however, only one credential is applied and a firewall used for protection may be broken [9]. Thus, the chances are that if the database is tampered with in any way, because there is no backup the data may be permanently lost [10]. Therefore, storing student records in a decentralized way, e.g., in the form of a blockchain ledger, alloys the above problem regarding a firewall [11]. Data cannot be lost because more than one copy of the blockchain is available. In addition, some universities store student data in a local machine without network availability, through which remote access to the data becomes more difficult, and thereby increasing the chances of forgery because no official records are available remotely for cross verification. The use of blockchain makes it easy to access student data remotely [12]. The system proposed in this paper operates in the following manner. First the educational institute/university uploads the student profile data consisting of the academic performance, extra-curricular activities, degree certificate, and other achievement certificates at the end of the course. A blockchain will then be created from these data. This blockchain will be used as a reliable data source to feed the ML algorithms, through which the students can be suggested a field of work more suited to them in the future. As another benefit, employers can rely on the data stored in the blockchain at the time of the recruitment process, which leaves the candidates with absolutely no chances to present any forged documents. In Related works section of this paper, related studies conducted in the fields of blockchain and ML are described. Methodology section covers the methodology of the proposed system, covering two important questions: (1) How does the proposed system operate, and (2) how valid is its operation? In Results and discussion section, the results and a discussion of the proposed system are provided. Challenges and future applications section describes the challenges in building this system as well as the future scopes of its application. Finally, in Conclusions section, some concluding remarks regarding the synergy between two blooming technologies applied to the proposed system are given.

## Related works

Throughout the educational field, numerous improvements have been suggested, among which a few are mentioned in this section. Various ongoing studies on blockchain and ML as independent technologies applied to this field are covered.

### Blockchain-based systems

The advancement of blockchain technology in terms of validation and data security has also been applied in the educational sector, with the validation and security of student data being considered prime aspects. Various systems providing such validation and security have been developed. For example, Li and Wu [13] proposed the idea of flashing the system, through which the counterfeiting of student degrees can be avoided. Such a program has to some degree remedied the defects found in current solutions, making the use of a blockchain-based certificate a more feasible theory.

Many types of certificates of success, grades, and diplomas, among other documents, can become a valuable tool in finding a new school or job. Gopal and Prakash [14] proposed a blockchain-based digital certificate scheme based on the immutable properties of blockchain. This scheme preserves the essential data and eliminates the chance of any company with a job offering distrusting a student’s certification. Individual learning records are important for the professional careers of individuals. Certificates support the achievement of learning outcomes in education. Furthermore, recipients can conveniently store paper certificates or display them to everyone and for whatever reason. Therefore, such records must be kept in long-term usable ledgers that are tamper-proof. Gräther et al. [15] introduced an alternative to paper certificates that were cryptographically signed. The system primarily assists authorities, learners, and employers in the verification process. This ensures productivity as well as protection for certification authorities by digitizing existing procedures, issuing and storing certificates in a database, and providing automated certificate monitoring, which will ensure the truthfulness of the certificates. However, more effort is needed to secure the registry for certificates and an open standard for digital signatures must be used; otherwise, a global verification of digital certificates will not be made possible. Various other studies on blockchain in education include the following: (1) The University of Nicosia used blockchain technology to handle student data, that is, certificates that they received from MOOC platforms; (2) Massachusetts Institute of Technology has advanced a blockchain-based learning machine technology and built a wallet for their students that contain their educational records; (3) Holberton School is also using this technology to save the educational record of its students, including their learning activities in the classroom, their identity, and their learning process; (4) Echolink is a blockchain platform at the international level that stores verified identity details, work experience, and skills in a hashed and immutable way. All information is inserted by legitimate institutions, and thus provides the trustworthiness of such information. Echolink sanctioned a partnership with Microsoft to offer a blockchain application cloud service on Azure; (5) Teach Me Please is a multi-functioning blockchain that helps in creating and storing personal and verified professional and academic profiles, thereby helping companies with job offers by providing a digital curriculum vitae of students generated based on their educational careers, accorded by credibility proofs; and (6) An application called Open Certificates assigns block-proof to educational certificates using smart contracts with Ethereum [16].

### ML-based systems

A similar development in the field of ML was observed, the main purpose of which is to predict the future occurrence based on past data, i.e., the available training data. Many studies have been carried out to predict the academic performance of students and their achievements of their major, as indicated in ref. [17]. The effects of this framework were tested in two phases. The first level is measuring the most commonly used metrics such as the mean absolute error, root mean square error, relative absolute error, and relative squared error, and the second level is applied by a pedagogical committee led by the school principal. Robotic process automation (RPA) is an important part of ML in the education field because it can collect large chunks of student-related data and provide them with an experience that suits their needs. For example, work fusion, an intelligent automation business, has an advanced platform called RPA Express that uses smart algorithms to determine which teaching methods are likely to work for each student [18]. Technological advancements such as this allow lecturers to assist students who may have an impairment or a particular learning experience to more effectively understand the concepts of their classes. The education sector provides a multitude of fascinating and demanding machine-learning applications. Students would like to select the best course based on how well they are predicted to do in the courses chosen [19]. With so much knowledge available and so many different needs, it is reasonably foreseeable that, particularly in the 21st century, an integrated ML system capable of meeting the special needs of an educational institution will be in great demand. Kotsiantis [20] aimed to fill in the gap between a factual student performance prediction and current regression techniques. This study used current regression methods to predict student outcomes. Some regression algorithms were compared to determine which is more appropriate, not only in reliably predicting student outcomes but also as an educational support tool for tutors [21–23].

Today, proper guidance for first-year students in universities is considered extremely important. Dekker et al. [24] described the results of an educational data mining case study aimed at electrical engineering students dropping out after their first semester or even before they start their courses. This case study was aimed at the Electrical Engineering Department of the Eindhoven University of Technology. The experimental results show that simple and intuitive classifiers provide useful results with accuracies of between 75 and 80 %. Supervised learning was applied to the dataset of students in their first year in the department, and a new approach to labeling was determined. A student with a diploma was classified as successful. The authors used several tools and techniques such as Waikato Environment for Knowledge Analysis classifiers in their experimental study and compared two decision tree algorithms, C4.5(J48) and classification and regression tree (CART, SimpleCart), as well a Bayesian classifier, a logistic model, a random forest, and a rule-based learner [25]. They even considered the OneR classifier as an indicator of the predictive power of attributes and a baseline.

The aforementioned studies stated the use of blockchain or ML as independent technologies. Therefore, systems containing blockchain as a prime technology are unable to demonstrate the extra benefits that can be made available through a prediction using ML and whereas a system with ML as the prime technology lacks in the ability to demonstrate data security. Hence, by considering the merits of both ideologies, a system is proposed that eliminates the demerits stated above and converts them into the main advantage only system with the help of synergism between ML and blockchain technology.

### Blockchain and ML integration

In a previous review paper, the authors presented a basic outline of how to integrate blockchain and ML [26]. In this paper, the proposed system is implemented in three phases: the ML phase, which includes the proposed model for predicting the future job roles of students, a blockchain phase in which blockchain is implemented from scratch, which works as a database to store student records rather securely, and an integration phase that merges the ML classifier and blockchain, allowing them to work in harmony. The next section illustrates the operation of the proposed system.

## Methodology

Universities are now seeing blockchain as a secure medium to store and maintain the academic records of students because blockchain is a transparent, distributed, and unmodified ledger [27]. This shared and unmodifiable database prevents document forgeries and provides a simple and quick way to look at student credentials. The direct link between students’ digitally stored academic information will save time and wipe out the paper-based system of information sharing, which may involve forged documents. This system provides a full image of a candidate’s skills and background. Employers do not have to contact the college to verify the credentials of the candidate.

**Fig. 1:**
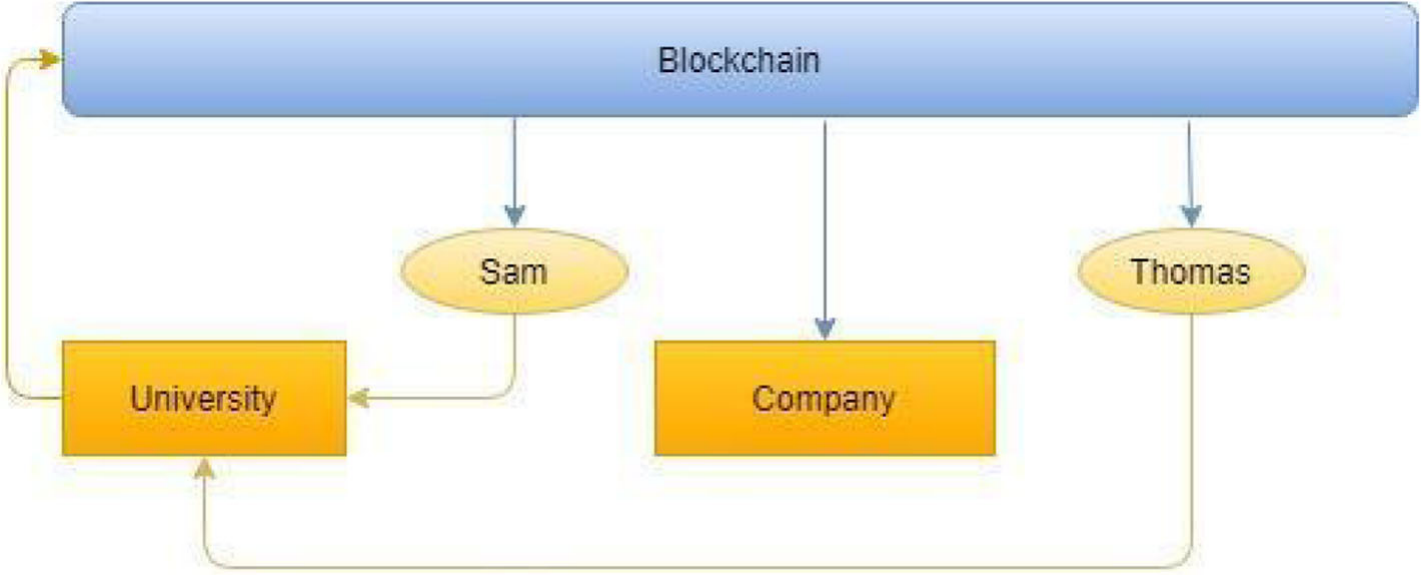
Describing the role of the stakeholders and their interaction in the proposed system. Sam and Thomas are university students

In the proposed system, merging the capabilities of blockchain and ML to advance the education sector is proposed. The authors have considered various stakeholders in managing and accessing educational records, including students, company employers, universities, and authorities, to ensure that the proposed system caters to what is needed for security, easy accessibility, and availability. Figure 1 describes the various stakeholders and their participation in the system.

Blockchain at the university level is needed because of the increasing rates of forgery and the generation of fake degrees. The university should have a standard authenticated and decentralized database that can store all records of the students who have graduated from the university. These academic data should not be manipulated or deleted by any outside influence, thereby ensuring the credibility of the system. After passing the final exams of the university, student data should be added to this decentralized database, which includes the grades of the main subjects of the respective major of the student, e.g., computer engineering, as well as the final cumulative grade point average (CGPA), number of backlogs, number of projects conducted by the student throughout the course, extracurricular activities under any faculty such as online quizzes, and athletic achievements. None of the data should be manipulated, and the data must be securely stored without loss. With blockchain, which is a decentralized database, there may be more than one copy of the data, and therefore if one chain is affected, other data chains are available. Academic data, as described above, can be added to a blockchain as a block with each student enrolment number as a unique identifier, which assists in tracking transfers between two anonymous parties to any traded digital object. Blockchain-supported security components are crucial for maintaining accountability, anonymity, and fraud prevention. The following are the high-level authentication features of a blockchain.

1. Ledger: In the blockchain, the ledger logs each transaction. The ledger is a block chain, and the data are immutable in the block. The delivery of the ledger to all nodes was completed.
2. Chain of blocks: Each block has a hash value of the previous block, which forms a chain. Block data corrections (*n*) change the hash value and do not validate the hash contained in the next block (*n* + 1). This is a reaction of the chain that influences the overall chain. This characteristic strengthens the security of confidential data and knowledge.
3. Confidentiality: Blockchain promises secrecy by allowing users of a database to see only approved transactions.
4. Transparency: By exchanging the ledger with all nodes and using consensus algorithms to achieve consensus among all nodes, blockchain enables transactions and the ledger state to be stored and handled transparently. The ordering and execution of transactions are often guaranteed by consensus algorithms.
5. Cryptology: This allows encrypted transactions and renders the blockchain immutable using hash-based algorithms that generate a fixed hash based on the content of the block.
6. Smart contracts: A machine code running on top of a blockchain containing a series of rules, the basis upon which the parties choose to communicate with each other, is a smart contract. If the predefined rules are followed, then the arrangement is automatically executed. No contract can be executed without the consensus of the network.

Until now, the security aspect of the system has been ensured. The second component of the system was ML. ML in the education system, as discussed in the previous section, is highly beneficial. However, what if the data being entered into the model for prediction are not valid but are instead fake? Further, what if the data the model is being trained on has not been validated? In such cases, nothing more than a garbage in garbage out process would be applied. In the proposed system, focus is mainly on providing valid data to the ML model to provide accurate and validated predictions. In this case, data is stored in the blockchain such that no third party will have any influence over them. In addition, it was ensured that the system does not lose the educational records of the students. Now, predictions will be made about the type of job students should find after graduation. In this way, a student can obtain accurate suggestions for the job the student should pursue based on past academic data. For this, student data from the blockchain are directly fed to the ML model for training and prediction.

**Fig. 2:**
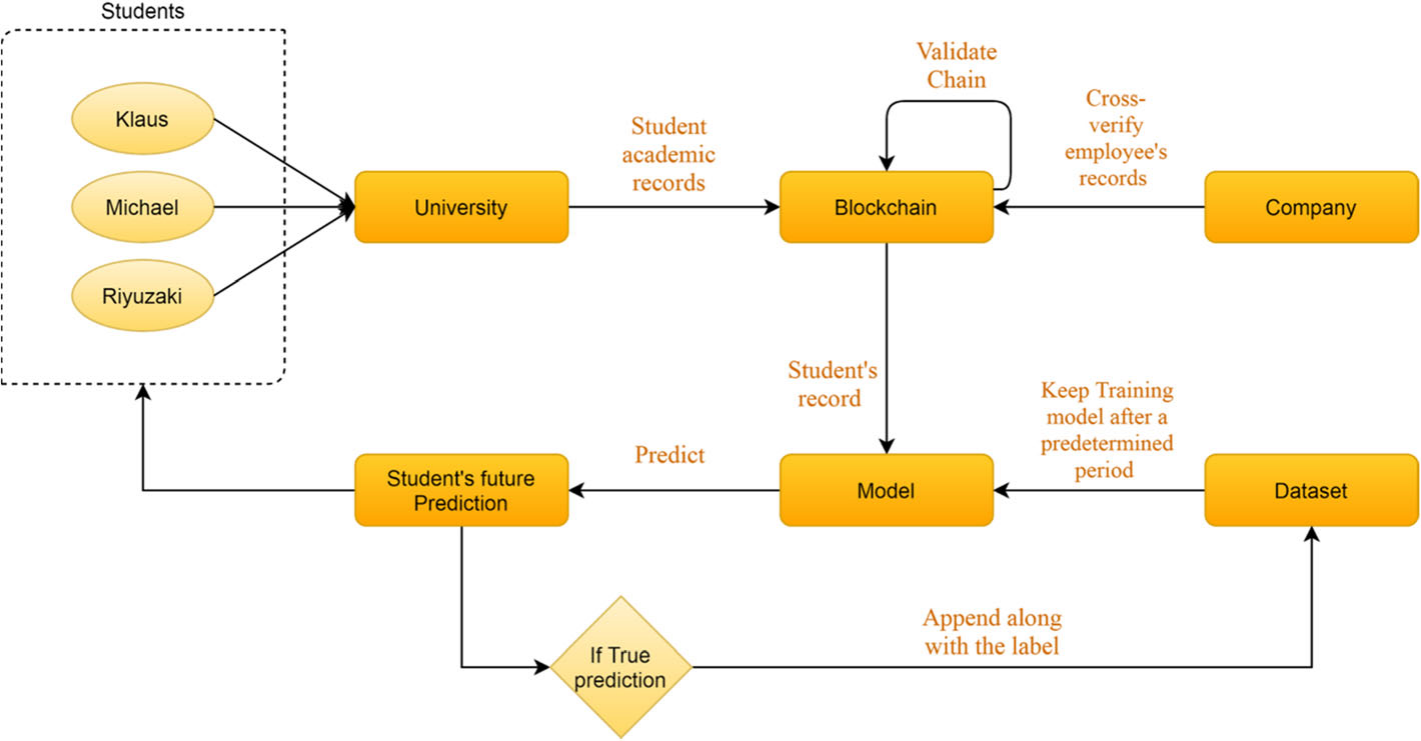
Flow of the proposed system

As the third component of the system, employers will benefit from this system. Such employers will no longer have to worry about the credibility of the records the employee is representing. Trusted companies can have access to the blockchain of the university to verify the records of graduating students. This will add the task of the blockchain data verification into the recruitment process of such companies. In addition, students in the future can share their credible data on any platform, including LinkedIn, which is a platform for managing one’s professional identity. Such data will be referred to as the official record of the students. Figure 2 shows the flow of the proposed system discussed above.

**Fig. 3:**
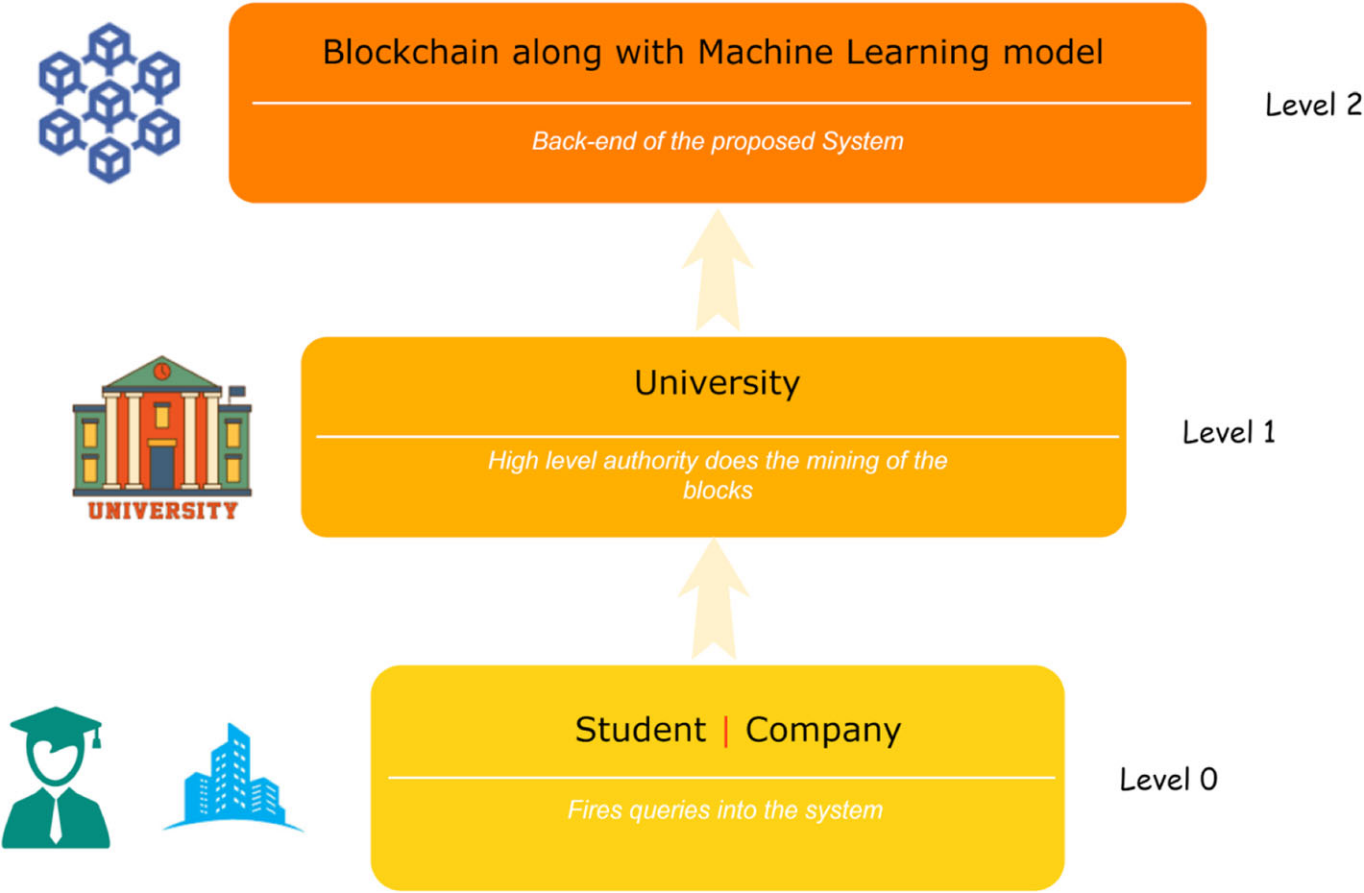
Levels of hierarchy in proposed system

For a better understanding of the system and its hierarchical pattern of the stakeholders, the system is described in a hierarchical form, which includes the levels containing a specific set of stakeholders and their tasks relevant to the level. Figure 3 shows the hierarchy of the proposed system.

### User-level (Level 0)

This level is called the user level because it describes the interacting entity of the system. This classification of interacting entities helps in understanding the demographics and taste of the user by which the system can be made more user friendly. This also helps define the proper layout of the system because it identifies the segment of the various types of users of the system as students, companies, or other entities. Here, students and companies can view the data stored in the blockchain by firing queries into the system.

### Intermediate level (Level 1)

This level describes the authority that manages the system and plays the role of a system mediator. The role of mining as well as computing the proof of work of the block containing the student data is conducted by an intermediate level team, which is the managerial authority. This team also looks after the maintenance of the system and saves it from malfunctioning. Thus, the intermediate level can be considered a glue that helps the user stick with the system.

### Processing level (Level 2)

This level contains a decentralized database. At this level, the processing task of the query fired by the user is performed. The user request is processed, and the result is provided by this layer. This is the most crucial part of the system, and a malfunction of this layer may bring the system to a halt. Therefore, utmost care should be taken when handling it. Many types of backend software such as Ethereum virtual machine serve at this level.

## Results and discussion

This section covers three important subsections to help users navigate through the proposed system. The first is the ML part of the proposed system, which contains a description of the dataset that was used and shows the accuracies of the various models applied. The second subsection describes the blockchain component of the system, which is guided through the structure of the blockchain in the proposed system. Finally, the third subsection is the most important, and describes the integration of both technologies.

### ML phase

The first step in any project based on ML is to collect data concerning the relevant domain. The dataset was created by surveying 1540 students who had graduated from the Computer Department of Vishwakarma Government Engineering College, India, with the help of a Google form consisting of various questions regarding their academics. The dataset contains features such as student marks in the various important subjects of computer engineering, which are scored on a scale of up to 100. At the end of the survey process, the data contained 22 independent features and one dependent feature that contained categorical values. It contains the CGPA of the students and other features such as marks of the 10th and 12th standards and participation of the student in the technical quiz, technical project, rank, gender, number of backlogs, miscellaneous technical events, and number of athletic achievements. The dataset also has features stating the board name of the 10th and 12th standards that the student went to. The dataset used has a total of five categorical features that need to be further reduced to numerical features by label encoding or one hot encoding while applying a particular ML algorithm. In addition, it has eight discrete numerical features, and the remaining continuous numerical features. The dependent feature in the dataset is the job of the student. This is the target feature, which is dependent on the remaining features. The dataset is created while taking into consideration the data that might be from the official records of a student at the university.

**Fig. 4:**
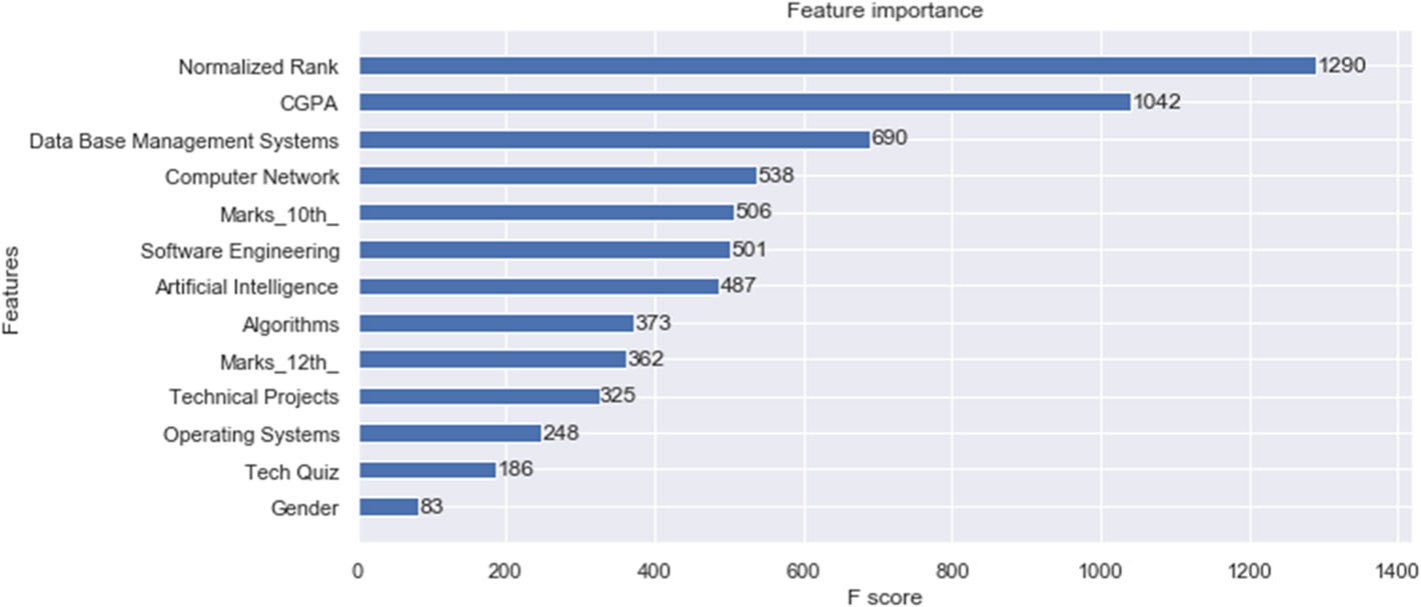
Feature importance of the dataset

**Fig. 5:**
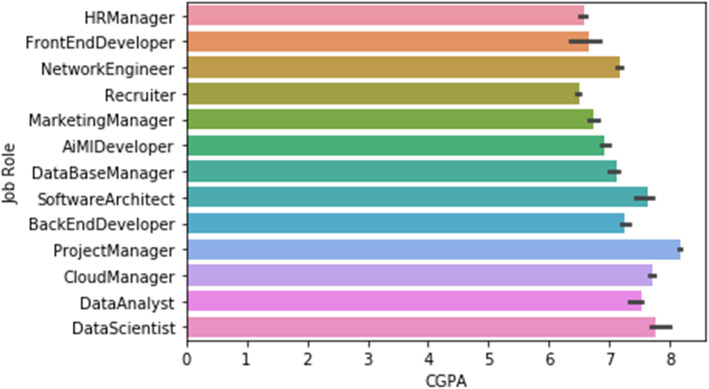
Barplot (job role vs CGPA of students)

Figure 4 shows an exploratory data analysis, and indicates the feature importance in which the F score along the X-axis is called the “feature score”, which is a metric simply summarizing how many times each function is split. The normalized rank is the most important feature of the dataset. The CGPA being the second most important feature on the dataset provides a better understanding of how the job role is affected by it. Figure 5 shows a seaborn barplot (job role vs CGPA). Error bars in the bar chart are used to show where each bar can be while taking into account any errors. In the chart below, the median aggregate is used for the error bars.

**Fig. 6:**
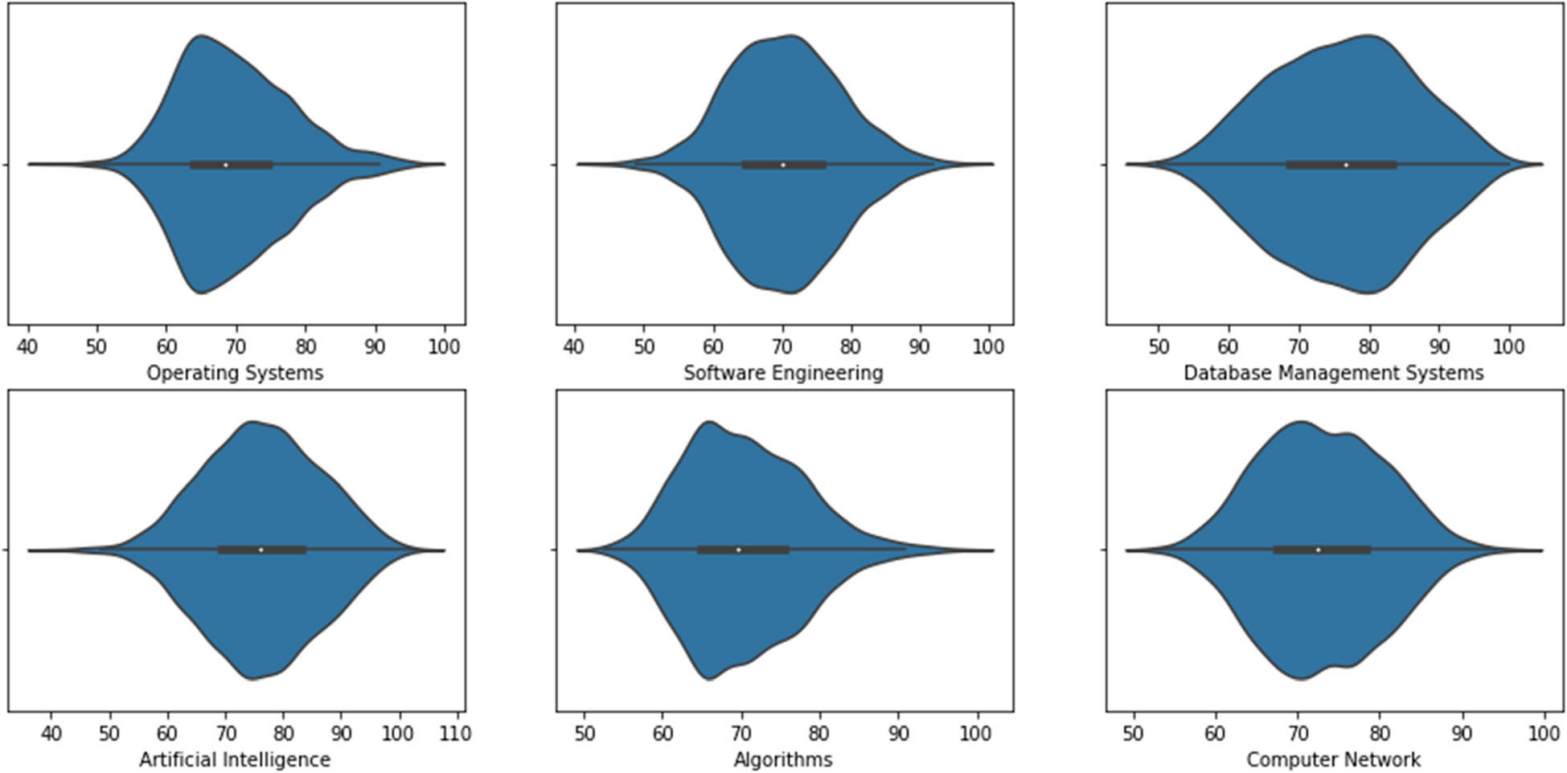
Violin plot representing the distribution of marks of the main subjects

The distribution of marks of the students in the dataset is explained with the help of the seaborn violin plot in Fig. 6. Violin plots are best for representing the distribution of data because they provide an estimation of the dataset instead of mapping each data individually. In the violin plots, there are two symmetric kernel density estimator plots along the centerline. The white dot on the line represents the median, whereas the thick line indicates the interquartile range at the center of each violin. The line extending from the thick line to the ends showed a confidence interval of 95%.

Further, many ML classifiers were tested on the dataset to predict the job role based on the academic records of the student, where Table 1 shows the accuracies of the different models tested for the datasets applied.

**Table 1:** Classifier accuracy on student dataset

Classifiers	Perceptron	Decision tree	SVM	Random forest	Extreme Gradient Boosting	KNN	Logistic regression
Accuracy	0.66	0.86	0.92	0.88	0.93	0.86	0.75

From the above models, the support vector machine and Extreme Gradient Boosting (XGBoost) yielded the best results. Below is a brief description of these two models: (1) Support vector machines: This is a powerful ML tool that makes classifications by defining decision boundaries, also called a “decision hyperplane” herein, between classes and then sees what side an unclassified point lies on. (2) XGBoost: This is a boosting method used for ensemble learning. It provides more accurate results than any other model trained because of the boosting method it uses.

### Blockchain phase

**Fig. 7:**
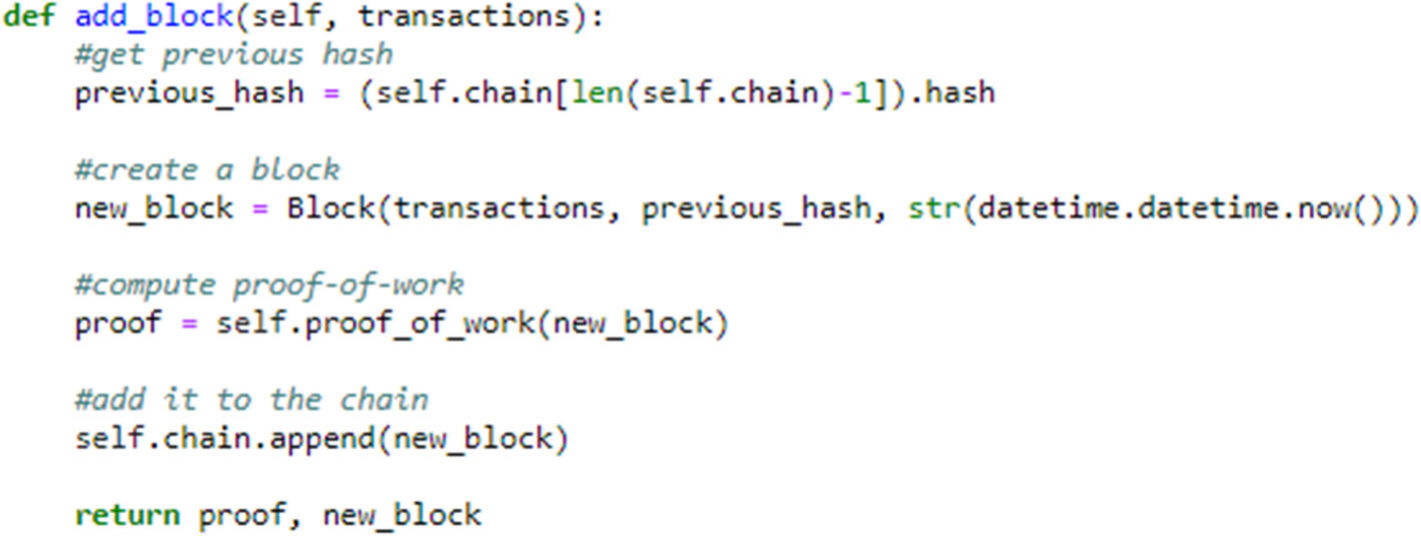
Blockchain class method for adding student’s blocks to the chain

To represent a blockchain and block, Python classes are used in which the methods and member variables were initialized. In the blockchain class, there are many methods for various tasks, such as adding a block, validating the chain, and computing the proof of work. Likewise, in the block class, there is a method for computing the hash of the block using the secure hash algorithm 256 (SHA256) method of Python. Figure 7 shows a code snippet of a simple method for adding a block to a chain. With this method, the transactions, which in this case are the student data, are passed in the form of a Python dictionary, and a new block is created and appended to the chain.

**Fig. 8:**
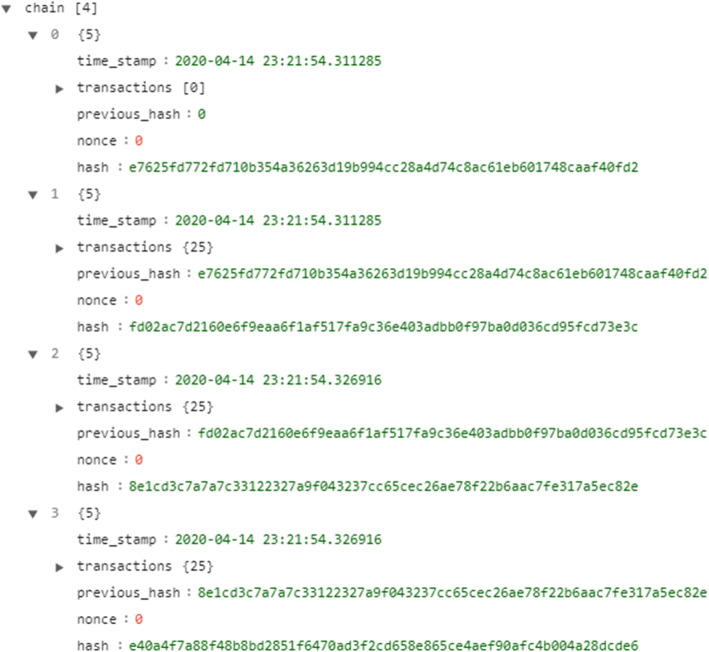
Blockchain consisting of three blocks of students and one genesis block

An example of the blockchain of the proposed system is shown in Fig. 8, which contains three blocks of students and a genesis block. Each block inside the ‘chain’ is stored as a Python dictionary. The chain itself was defined as a Python list. The first block in the chain is always a genesis block that does not have any data and has a previous hash of zero. For the remaining blocks, the student data are stored inside the transaction property as a Python dictionary. Each block contains a timestamp, which is the time when the block is mined to the chain. The previous hash inside the block must match the hash of the previous block. Hashes in the blocks are SHA256 encrypted, and these hashes are created using all data in the block. These hashes, however, are calculated using a proof-of-work algorithm in which the hash of the block must start with a number of zeros, which is called the “difficulty”. In the Bitcoin blockchain the ‘difficulty’ is 12. A nonce is a value used in computing the proof of work, and is incremented and added to the data until the desired number of leading zeros appears. If any data are to be altered in any block, then its hash value will also be altered; thus, the previous hash value of a successive block will no longer match, and the chain will be broken.

**Fig. 9:**
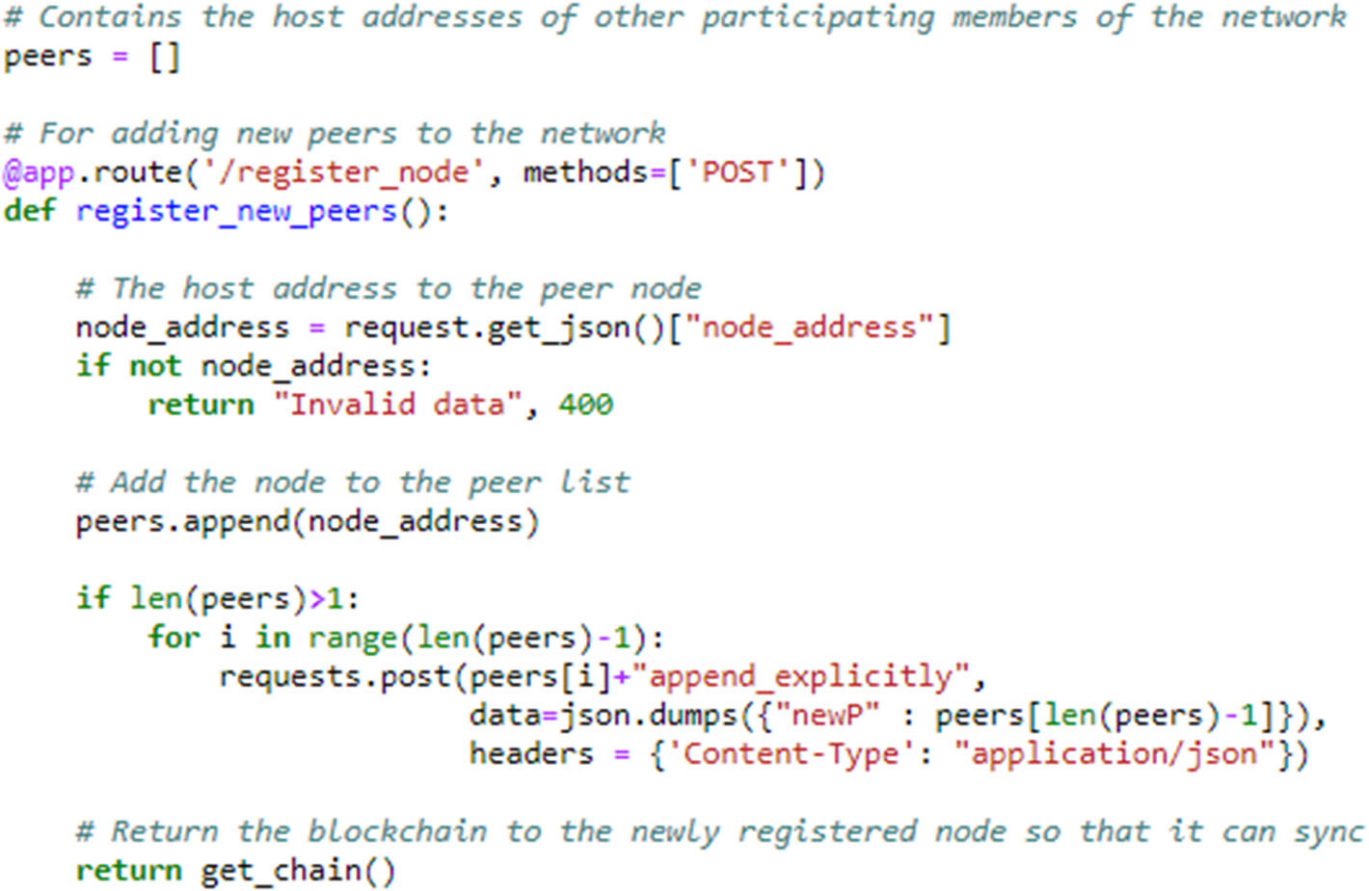
Code for registering a new node to the network

Furthermore, the authors attempted to establish decentralism in the system. To do so, a function was created that allows this to become a peer-to-peer system from a single-node system. Figure 9 shows the snippet of a function or flask router to make a new participant within the network. This endpoint of the flask server receives a post request from the new-to-be node and takes a node address in the query to append it to the list of known nodes or peers in the network. Furthermore, the server that receives the request sends a request to other participants in the network to update their list of peers. Finally, it returns the chain to the node that sent the request, with the help of a “get_chain()” function, which returns the copy of a chain from the already running blockchain. Thus, the new node can synchronize with the chain.

Now consider a case in which there are four participants in a network; thus, four blockchains, all having the same blocks, are applied. This time, adding the blocks to the chain will be different from the case in which there was only one participant. Here, a new block will be announced to all four participants first, and each of them will then start computing the proof-of-work. Whichever participant computes the proof-of-work first, adds the block to the chain, and the rest of the participants will then have to stop computing the proof-of-work. After adding the block to the blockchain, the participant sends requests to other participants to update their copy of the blockchain accordingly.

### Integration of blockchain and ML

This is the final experimental phase of the research focused on here, combining both of the above-stated technologies and setting up a basic framework on how to process the data coming from blockchain through ML.

**Fig. 10:**
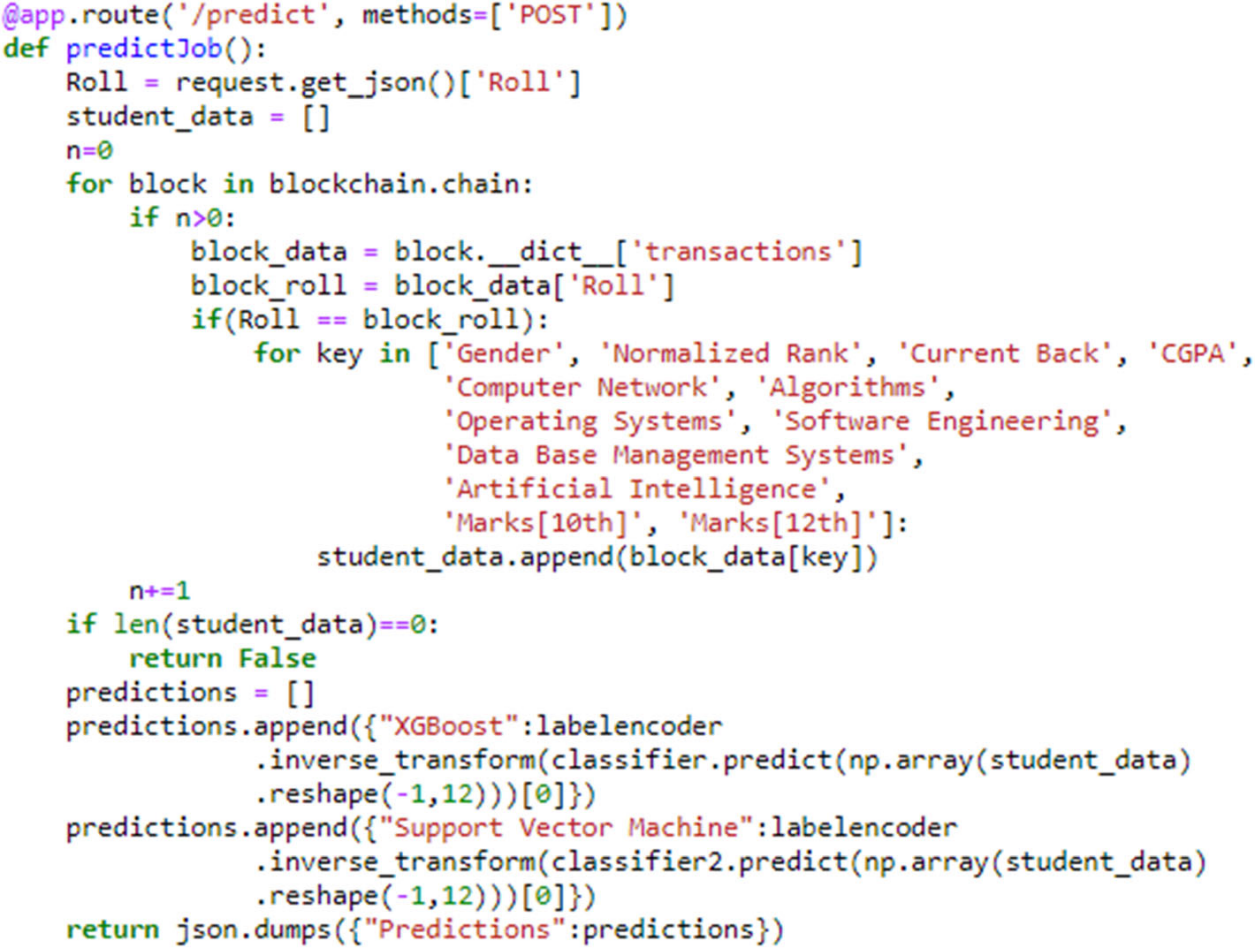
Implementation of the ‘/predict’ router of the Flask server upon which the blockchain and ML model is running

Figure 10 shows the code snippet of the application programming interface (API) that serves requests for predictions. The request is sent to the Flask server upon which the blockchain is running. Thus, the blockchain running on the server provides data to the ML model. In the code below, the variable ‘blockchain’ is the blockchain class. The blocks were added as described above. The data of the students are stored in a ‘transactions’ variable as a dictionary. The data of the students were retrieved from the block and stored on the “student_data” list. This list contains only feature values that are to be used to obtain predictions. The two best models were used for making predictions, i.e., a support vector machine and XGBoost, and a label encoder was applied to manage the categorical features.

**Fig. 11:**
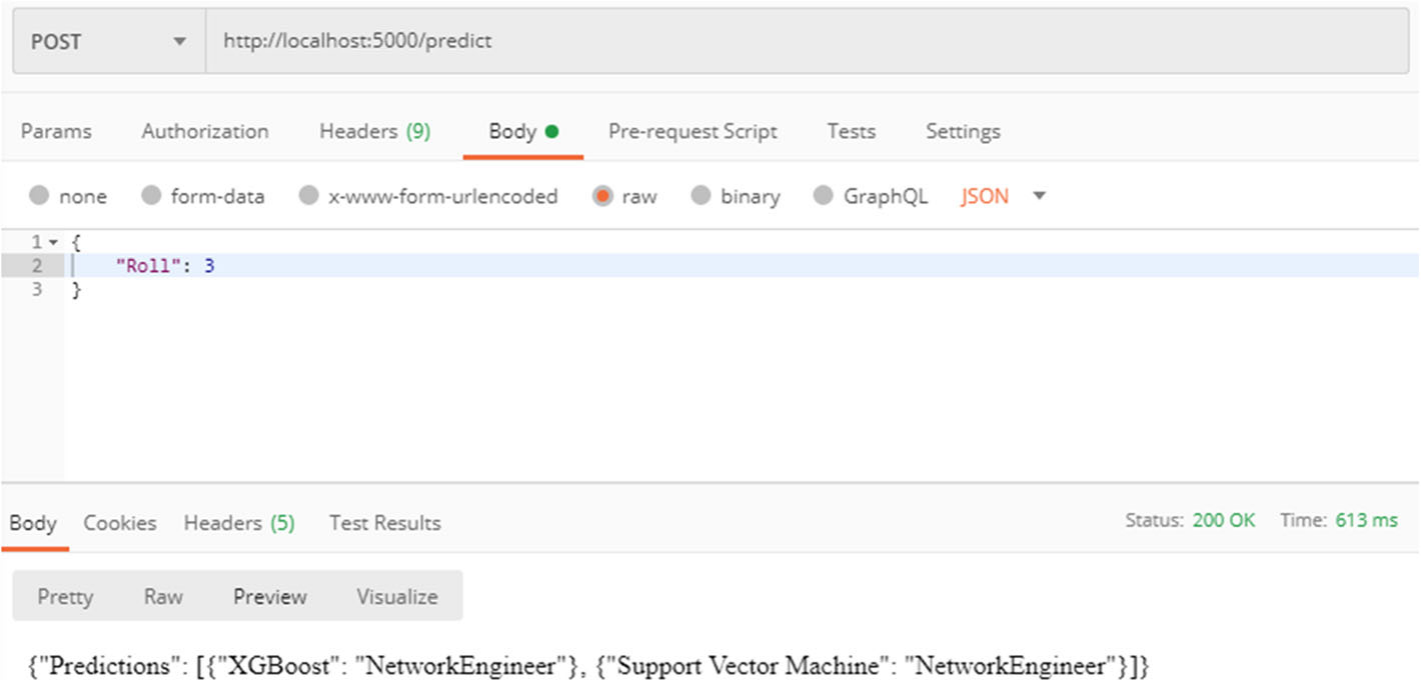
A ‘POST’ request made to the ‘predict’ router of the Flask server with the help of Postman software

Figure 11 shows the result of calling the API shown above. Here, for an easily understandable example, enrollment numbers in single digits were considered. This displays the operation of the experiment under a real-world scenario. However, in real scenarios, this number might be ‘170170107094’ which is unique to a student. Here, after sending a post request to the Flask server API, the json response is obtained, which is the prediction from both of the models trained. This integration of both technologies provides a new perspective on security for a traditionally operating centralized database system. The data from a truly legitimate source, such as blockchain, also provide novelty for the accurate and verified data available for model training.

## Challenges and future applications

First, blockchain technology used for education is still in the study phase and can be extended. To have a meaningful impact on the educational space, blockchain needs a transition at the base level, as well as cooperation from all stakeholders across boundaries. As a structured method, teachers subjectively need to study certain learning habits and learning outcomes. Without human involvement, it is extremely difficult to test this type of learning activity using a pre-programmed smart system [28, 29]. It should be noted that certain educational institutions are either hesitant to share their data with the blockchain network, or they find it difficult to determine which data to supply through the blockchain network. The compilation of records on blockchain from various institutes is a significant task.

Second, one of the main challenges in this system might be the retrieval of a block of students based on their enrollment number. The authors developed the chain as a Python list in which the blocks are added as Python dictionaries. For the proposed system to predict a student’s future job based on the student’s academic records throughout a course, the block belonging to that student needs to be fetched from the chain. In the proposed system, a for-loop is used to iterate over the whole chain and match the enrollment number of each block with that from which the data are currently wanted. After obtaining a match, the data are extracted and fed into the SVM and XGBoost models. As can be seen, the time complexity for this task is O(n), which is O(n), where n is the length of the chain. Although a limited number of blocks have been applied to explain the system, under a real-world scenario, the length of the chain may become extremely long as new blocks of graduating students are added every year. There are other ways to efficiently loop over the chain, for example, parallel programming.

Further, as shown in the Methodology section, after obtaining predictions from the ML model, the data along with a label can be appended to the training dataset if the prediction is true. The total overview of the discussed system can be described well by conducting a strength weakness opportunities and threats (SWOT) analysis.

**Fig. 12:**
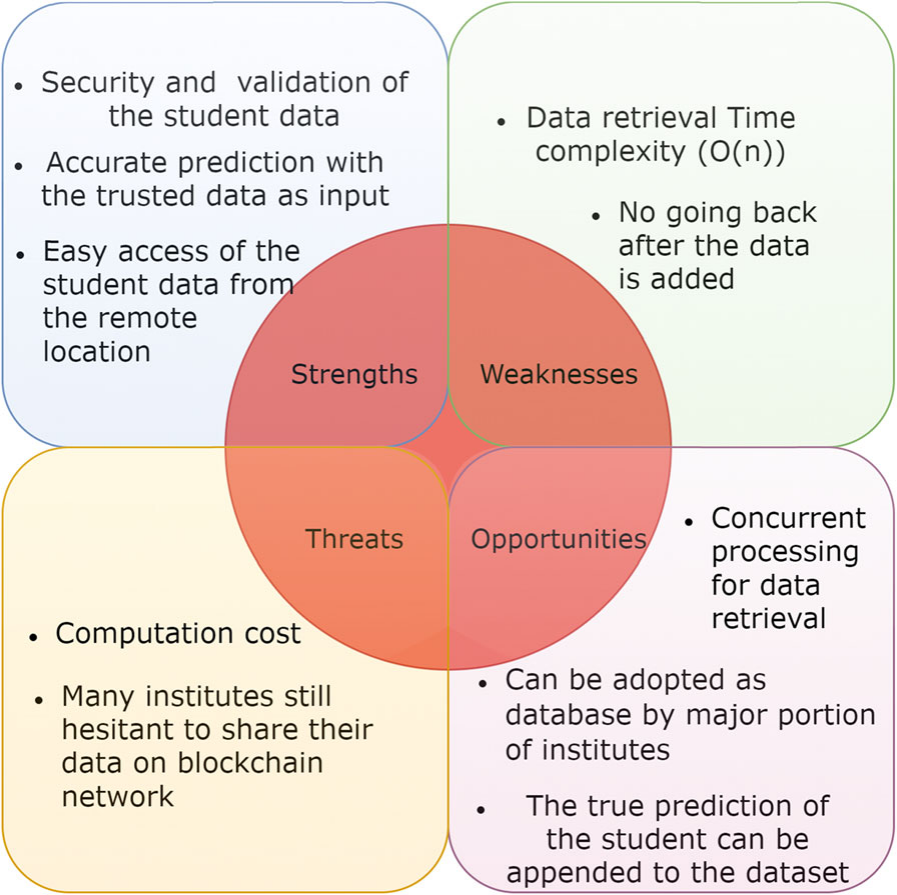
SWOT analysis of the system

A SWOT analysis of the proposed system is described in Fig. 12, which shows a summary of the entire system covering the various aspects through which a better identification and conclusion regarding the system can be obtained. The reason for conducting a SWOT analysis is to present the pros, cons, and overall scope of the system.

## Conclusions

When going through various technologies used in the educational discipline for the betterment of students, it was noted that the use of blockchain technology has worked successfully in mitigating the risk of forgery of student achievements, and that the use of ML provides students with predictions about future occurrences. Therefore, in this paper, a system was discussed that combines blockchain and ML technologies, providing the user with the benefits of both approaches. In addition, using ML on a trusted dataset validated through blockchain helps increase the veracity of a machine-learning-based model. This paper described how a university mines the blocks of the students and provides proof of work. This ML model can obtain the input of the trusted data and is capable of achieving a more accurate prediction. Hence, the main aim of this paper is to represent a system containing a blockchain that manages the student data in the form of a decentralized database (ledger), in which a job placement company can inquire about the student data directly on a blockchain and obtain the validated and verified data of a student extremely quickly, thereby obtaining accurate predictions regarding a suggested job for the student with the help of a trained ML model.

## Data Availability

All relevant data and material are presented in the main paper.

## Abbreviations

MOOC: Massive open online course
RPA: Robotic process automation
ML: Machine learning
CART: Classification and regression tree
CGPA: Cumulative grade point average
XGBoost: Extreme Gradient Boosting
SHA: Secure hash algorithm
API: Application programming interface
SWOT: Strength weakness opportunities and threats
